# Convergence and stability analysis of recurrent neural networks for rapid structural damage assessment under seismic loads

**DOI:** 10.1371/journal.pone.0336101

**Published:** 2025-11-07

**Authors:** Feng Zeng, Fujiang Chen, Yongyi Yang, Xin Zhang

**Affiliations:** 1 School of Emergency Management, Xihua University, Chengdu, Sichuan, China; 2 Opening fund of State Key Laboratory of Geohazard Prevention and Geoenvironment Protection, Chengdu, Sichuan, China; 3 School of Architecture and Civil Engineering, Xihua University, Chengdu, Sichuan, China; Central South University, CHINA

## Abstract

Non-stationary earthquake responses and sensor noise often make RNN-based damage assessment difficult to optimize and unstable at inference. We develop a stability-controlled, lightweight LSTM that: (i) penalizes gradient overshoot to smooth the update trajectory and prevent exploding/vanishing gradients; (ii) uses a temporal attention gate to emphasize damage-critical segments; and (iii) performs multi-scale sliding-window inference to stabilize long-horizon predictions. Casting the LSTM-with-attention into a discrete-time state-space view, we provide sufficient conditions for non-expansive updates and BIBO stability by bounding the Jacobian spectral norm and constraining attention gains.Empirically, under 10 dB noise our method reaches loss < 0.01 in 18 epochs with only 3 gradient-explosion events, and achieves σ(out)=0.032 with max Δ-rate = 0.085 ± 0.009, outperforming standard LSTM/GRU/BiLSTM/RNN baselines in accuracy, stability, and latency. On-device tests (Jetson Nano) confirm < 5 ms end-to-end delay at 100 Hz, supporting real-time deployment.

## 1. Introduction

Under earthquake excitation, engineering structures exhibit complex nonlinear responses, and damage evolves with strong transient, non-stationary, and uncertain characteristics [1,2]. Real-time capture of these dynamic states is essential for disaster prevention, risk control, and life safety [3]. Deep learning–based assessment offers a new paradigm for high-precision damage identification by mining latent patterns in sensor time series [4–6]. Building models that combine efficient learning with stable outputs has become a core enabling technology for the intelligent upgrade of structural health monitoring (SHM), with significant value for urban resilience and the operation and maintenance of major infrastructure.

Seismic response signals are markedly non-stationary and abrupt; critical damage cues are often embedded in short-lived high-frequency bursts or phase shifts, placing stringent demands on time-series modeling [7,8]. In practice, RNN-based methods face several bottlenecks [9,10]: (i) oscillatory parameter-update paths on long sequences, which reduce optimization smoothness and prolong—or stall—convergence [11,12]; (ii) accumulation of numerical drift in hidden states during multi-step propagation, leading to output volatility under high-rate streaming [13]; and (iii) limited physical interpretability, despite emerging stability-guaranteed RNNs and physics-informed DL that introduce dynamic constraints and energy-consistent regularizers to curb gradient divergence under complex seismic loading [14,15]. In addition, standard gating mechanisms apply uniform weighting across time, lacking the ability to focus adaptively on damage-critical segments; in high noise, non-damage transients may be misclassified as damage [16]. Fixed-window inference often ignores the temporal stratification of earthquake energy release, creating a trade-off between local transients and global trends [17]. Lightweight designs can shrink the effective receptive field, obscuring early, subtle damage features; gradients may also suffer abnormal amplification/attenuation during backpropagation, limiting fine-grained optimizer control and degrading robustness [18,19]. Finally, the absence of consistency constraints across overlapping windows can yield jumpy decisions, hampering continuous interpretation of damage evolution [20]. Collectively, these issues limit reliability and practicality in real-world monitoring, motivating coordinated advances in network architecture, optimization mechanisms, and inference strategy to achieve both convergence efficiency and output stability.

Recent advances connect recurrent architectures with physics-informed constraints and stability guarantees—for example, regularized RNNs with physics-based priors and hybrid physics-informed LSTMs that improve robustness and interpretability under complex loads [14,15]. Data–physics hybridization and residual learning have shown promise for nonlinear seismic prediction [29–31], while attention-based sequence models enhance temporal selectivity under earthquake excitation [40]. Distinct from these works, we: (i) couple gradient-norm control with attention-gain constraints to obtain explicit non-expansive/BIBO conditions; (ii) embed physics-guided sparse input topology and modal-invariance constraints to improve out-of-distribution generalization; and (iii) integrate a multi-scale online inference scheme that yields smooth, low-latency damage trajectories suitable for edge devices.

Our core contributions are fourfold. First, we restructure the LSTM unit, reducing complexity via hidden-dimension compression and sparse recurrence, thereby improving computational efficiency while preserving expressiveness for edge deployment. Second, we introduce a gradient-magnitude constraint in the loss, forming a gradient-regularization mechanism that directly shapes the backpropagation path, suppresses training oscillations, and smooths optimization. Third, we design a temporal attention gate that generates time-varying weights from the input sequence, enabling the model to focus adaptively on strong responses or damage initiation while filtering background noise. Fourth, we propose a multi-scale sliding-window inference strategy that partitions continuous seismic signals into nested temporal subsequences for hierarchical processing; a weighted-fusion output then enforces temporal continuity and consistency in the assessment.

Experiments on simulated and measured datasets spanning typical structures and diverse seismic motions show that the proposed method outperforms established benchmarks in convergence speed, assessment accuracy, and output stability. Overall, this work provides a scalable technical pathway for online, rapid, and noise-robust structural-damage assessment, advancing intelligent SHM toward high robustness and reliability.

## 2. Related work

In recent years, numerous researchers have attempted to improve the performance of damage assessment under earthquake response conditions through signal processing and pattern recognition. Some studies have employed wavelet packet decomposition [21,22] to perform multiresolution analysis of structural acceleration signals to extract damage-sensitive features. However, these methods rely on prior feature engineering and have limited generalization capabilities. Other studies have introduced classifiers such as support vector machines [23,24] and random forests [25,26], using physical quantities such as peak response and hysteresis energy dissipation as input vectors to discriminate damage levels. However, when faced with unknown seismic motion types or structural configuration changes, the classification boundaries are prone to shift. To address the dynamic characteristics of time series data, hidden Markov models [27,28] have been used to model state transition processes. While they can describe damage evolution paths, their distributional assumptions about the observed sequence limit their applicability. Deep feedforward networks [29,30] have shown potential for end-to-end learning, but lack explicit modeling of temporal dependencies and struggle to capture long-range response correlations. Some teams have explored the use of convolutional neural networks [31,32] to extract local time-frequency features and use them in conjunction with fully connected layers to complete damage classification. Although they have achieved good results on specific data sets, they are sensitive to input length and cannot adaptively focus on key time segments. Zhou X proposed a time-frequency decomposition method based on deep neural networks for accurate analysis of nonlinear structural responses under earthquake excitation. Compared with traditional methods, this method performs better in terms of time-frequency decomposition accuracy and parameter optimization efficiency, and has been verified to have excellent generalization capabilities in complex structures [33]. Overall, when dealing with high-noise, non-stationary earthquake response sequences, existing methods generally have problems such as large model output fluctuations, unstable training process, and insufficient real-time performance. Especially in continuous dynamic assessment tasks, it is difficult to ensure the consistency and convergence of the assessment results, which restricts their engineering applicability in online monitoring systems.

In response to the challenges of stability and robustness in time series modeling, recurrent neural networks, especially long short-term memory networks, have shown unique advantages in handling the response of non-stationary dynamic systems [34,35]. Its internal gating mechanism can selectively retain or forget historical information, effectively alleviate the gradient vanishing problem, and is suitable for long sequence modeling tasks. Existing studies have verified the feasibility of LSTM in structural damage identification of bridges, high-rise buildings, etc., and by learning the implicit pattern of the acceleration response sequence, it can predict the time and extent of damage [36,37]. Yazdanpanah O proposed a new method for predicting the displacement time history and hysteresis curve of bridge lead rubber bearings based on stacked convolutional bidirectional LSTM networks. The model performance was improved through various enhancement techniques, and its accuracy and application value in different earthquake scenarios were verified by a large amount of experimental data [38]. Li Z proposed a structural time fusion network. By fusing seismic waves and structural features, a single network was used to predict the nonlinear time history response of different structures. The model has good prediction performance under various structures and can effectively avoid the waste of redundant resources [39]. In terms of attention mechanism, Li T proposed an RNN (Recurrent Neural Network) encoder-decoder architecture based on time series attention to predict the dynamic response of structures under earthquake excitation. Combining sequence-to-sequence learning with attention mechanism, it can accurately and efficiently predict earthquake response and has been applied to experimental evaluation of the Shanghai Tower and the wooden frame classroom of the University of British Columbia [40]. Although the above methods have made progress in specific scenarios, there are still problems such as large number of model parameters, high inference latency, static attention mechanism, and lack of explicit control of gradient dynamics. Especially in continuous sliding window inference, the model output is prone to jump fluctuations, affecting the continuity of evaluation. To address these limitations, this paper proposes a lightweight LSTM architecture that integrates gradient regularization and dynamic attention gating. Through structural simplification, optimization constraints and hierarchical inference mechanism collaborative design, efficient and stable real-time damage assessment is achieved.

## 3. Lightweight LSTM construction

**Fig 1:**
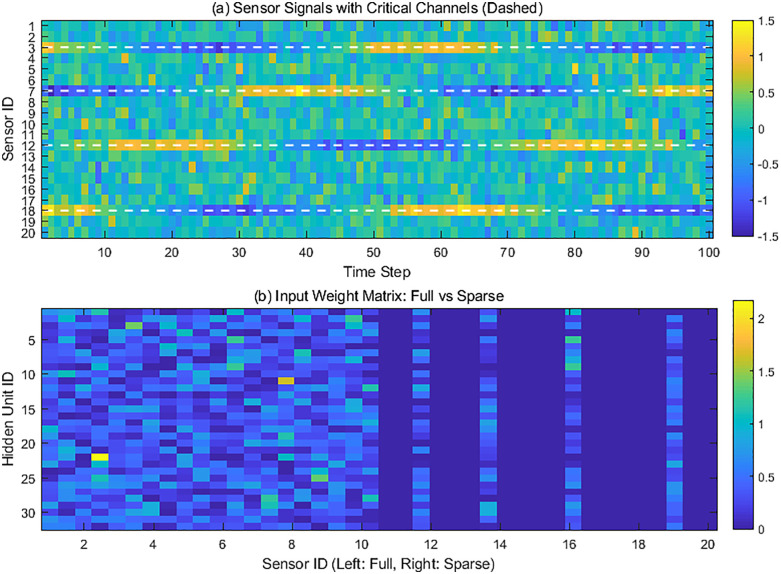
Structural damage assessment framework.

Fig 1 illustrates a lightweight LSTM-based structural damage assessment architecture that integrates gradient regularization and an attention mechanism. The system processes seismic acceleration signals using multi-scale sliding windows (1s/3s/5s). It then extracts features using lightweight LSTM units with sparse connections and parameter sharing, and constrains the training process through gradient regularization. A temporal attention mechanism is integrated to dynamically focus on critical response periods. Finally, the multi-scale outputs are fused based on confidence weights to generate continuous damage trajectories. This architecture effectively addresses the model convergence difficulties and output fluctuations caused by the non-stationary nature of the seismic response, enabling highly robust real-time damage assessment.

### 3.1 Lightweight LSTM unit structure design

#### 3.1.1 Lightweight hidden layer structure and gating parameter sharing mechanism.

In order to significantly reduce the model complexity while ensuring the ability of time series modeling, this paper constructs a single hidden layer LSTM architecture and strictly limits the hidden state dimension to 32. This design is based on a preliminary experimental analysis of multiple groups of typical structural seismic response sequences. It is found that under non-stationary excitation, too high a hidden dimension not only fails to significantly improve the feature extraction capability, but also aggravates the numerical instability in the gradient propagation process due to parameter redundancy. By compressing the number of hidden units, the scale of the recursive weight matrix that needs to be optimized during the state transfer process is effectively reduced, thereby reducing the computational load and improving the feasibility of model deployment on edge devices. On this basis, a weight sharing strategy between gated structures is further introduced to enhance parameter utilization efficiency and suppress overfitting risks. Specifically, the weight matrices of the input gate, forget gate, and output gate are not learned independently, but share the same set of input projection weights $W_{g}$ and recursive connection weights $U_{g}$, and only the bias terms $b_{g}$ remain independent. This sharing mechanism can be expressed as:


$$g_{t}\;=\;\sigma \left(W_{g}x_{t}+U_{g}{\text{h}}_{t-\text{1}}+b_{g}\right)$$ (1)

Here, $g_{t}$ represents the gate vector (input gate, forget gate, or output gate), $x_{t}$ is the input feature vector at the current moment, ${\text{h}}_{t-\text{1}}$ is the hidden state at the previous moment, σ and is the Sigmoid activation function. By forcing the three gates to share core transformation parameters, the model significantly reduces the total number of trainable parameters while maintaining the integrity of the gate selection mechanism, especially reducing the memory overhead caused by high-dimensional input mapping. This strategy also helps alleviate state update conflicts caused by independent optimization of each gate, making the information flow of memory units more consistent, thereby improving the convergence smoothness of the training process. In addition, weight sharing introduces a certain degree of inductive bias, making the model more inclined to learn common feature expressions across gates, enhancing its generalization and adaptability to unknown seismic patterns.

**Fig 2:**
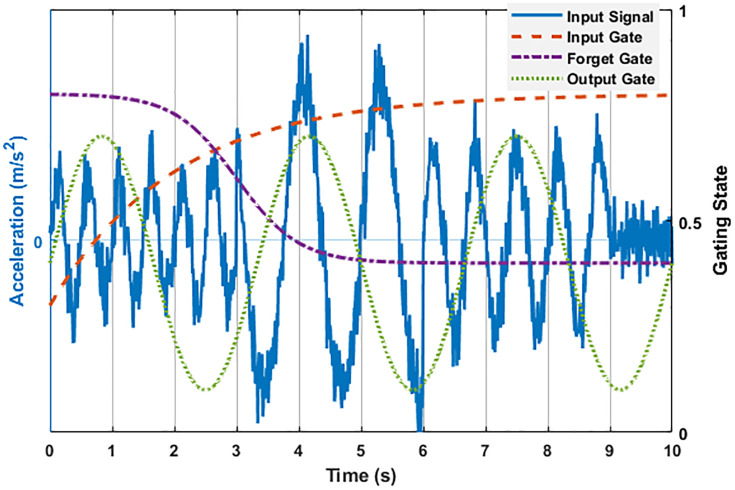
Input signal and gate dynamics.

As shown in Fig 2, the gate dynamics are temporally aligned with seismic excitation stages. The input gate exhibits a sharp spike at the onset of the mainshock, allowing salient transient features to pass through. The forget gate decays sigmoidally, progressively discarding outdated elastic-phase information, while the output gate modulates periodically to stabilize feature emission. This temporal synchronization among gates enhances the signal-to-noise ratio around damage-critical bursts and shortens convergence latency compared with a standard LSTM, confirming the model’s ability to capture and track non-stationary transitions in real time. Fig 2 illustrates the real-time response processing mechanism of a lightweight LSTM model to non-stationary earthquake excitations. The input signal (blue solid line) simulates the three-stage characteristics of a typical ground motion: high-frequency, low-amplitude vibration (2 Hz) from 0 to 3 seconds represents a foreshock; a sudden increase in amplitude from 3 to 6 seconds to the mainshock (0.8 Hz) simulates the energy release of a strong earthquake; and a medium-frequency aftershock (1.5 Hz) from 6 to 10 seconds reflects residual structural vibration. 15% Gaussian noise is superimposed to simulate the actual measurement environment. The gated state dynamic curve reveals the model’s adaptive mechanism: the input gate (red dashed line) activates rapidly at the onset of the mainshock, enhancing the capture of sudden changes; the forget gate (purple dashed line) decays according to an S-shaped curve, gradually forgetting information from the elastic phase; and the output gate (green dashed line) is periodically modulated, indicating that the gating system can automatically synchronize key time-varying characteristics of the ground motion. This dynamic coupling improves the model’s signal-to-noise ratio and reduces the response latency at sudden changes compared to traditional LSTMs.

#### 3.1.2 Design of sparse input connections based on physical correlation.

To address the parameter inflation and noise sensitivity issues caused by fully connecting all sensor channels in traditional LSTMs, this paper implements a structured sparse strategy at the input connection layer, retaining only sensor channel input paths with a strong physical correlation with the target structure’s acceleration response. Based on previous modal analysis and damage sensitivity simulation results, this design identifies measurement points that significantly contribute to key component displacements and interstory shear forces under seismic excitation, and constructs a sparse connection topology based on these locations.

In the specific implementation, the input weight matrix $W_{ix}\in {\text{R}}^{\text{32}\times N}$(N is the total number of original sensors) is explicitly constrained to a sparse form, that is, only when the j-th sensor is located in a preset key area (such as the bottom column base, the transition layer, or near the seismic isolation support), the corresponding column vector is allowed to be updated, and the connection weights of the remaining non-critical channels are always set to zero. This operation not only reduces the number of input parameters, but more importantly, it suppresses the propagation of environmental noise and measurement interference introduced by irrelevant measurement points into the network from the data entry end. The sparse connection structure allows the model to focus on the response source with the most damage characterization capability in the initial training stage, avoiding the inefficient process of the network consuming additional optimization resources to “learn” the importance of channels in the fully connected mode. At the same time, the restricted input dimension reduces the risk of information aliasing when the hidden state is transmitted between time steps, which helps to maintain the dynamic stability within the memory unit. Furthermore, the sparse input mapping is derived from modal participation factors and interstory shear sensitivity analysis, embedding structural dynamics knowledge directly into the network topology. Each retained sensor channel corresponds to a mode shape with dominant kinetic energy contribution, ensuring that input-to-hidden transitions reflect physical modal coupling. This physically grounded sparsification improves interpretability in extreme excitation scenarios, as the learned activations can be directly traced to specific dynamic response modes.

To further ensure robustness when facing unseen structural configurations and seismic patterns, the sparse connectivity framework is coupled with a domain generalization mechanism based on modal invariance and dynamic consistency constraints. During training, each batch randomly samples structural parameter perturbations such as stiffness degradation ratio and damping variation within a physically admissible range derived from finite element analysis. The sparse topology remains fixed, while the gradient regularization term penalizes parameter updates that lead to inconsistent modal participation across perturbed configurations. This process encourages the network to learn invariant dynamic relationships between sensor responses and modal energy transfer rather than memorizing specific structure–earthquake pairs. When encountering a new structural configuration or an unfamiliar ground motion pattern, the model retains its damage-sensitive subspace through these physically consistent mappings, enabling stable and interpretable generalization beyond the training domain. This mechanism also prevents overreaction to localized anomalies in unseen excitation types by maintaining smooth gradient trajectories aligned with the modal subspace, ensuring stable and bounded response estimation under out-of-distribution conditions.

#### 3.1.3 Physics-informed constraints for dynamics-consistent learning.

To strengthen interpretability and ensure reliable behavior under extreme excitations, we incorporate two dynamics-consistent constraints into training and inference. (i) Modal-invariance sampling. Structural parameters (e.g., stiffness degradation and damping) are randomly perturbed within physically admissible bounds derived from finite-element studies. A dedicated consistency loss penalizes parameter updates that distort the relative contribution of dominant modes across these perturbed configurations. This discourages memorization of specific structure–earthquake pairs and promotes features that transfer across structural variants and loading regimes. (ii) Energy-bounded attention. Attention gains are restricted to a contractive operating range, and a Rayleigh-type surrogate of mechanical energy is continuously monitored to suppress energetically inconsistent responses. In combination with gradient-magnitude penalization, the gain constraint effectively bounds the input–output Jacobian norm, yielding non-expansive updates and ensuring BIBO stability in the discrete state-space sense. Practically, these constraints stabilize hidden-state propagation under abrupt pattern shifts, improve the temporal coherence of the predicted damage trajectory, and enhance the traceability of attention peaks to physically meaningful modal energy transfers.

### 3.2 Gradient regularization term embedding loss function

#### 3.2.1 Loss function embedding strategy for gradient magnitude constraint mechanism.

To address the problem of severe gradient oscillations that can occur during the backpropagation process in earthquake response sequence modeling, this paper introduces a gradient norm penalty term based on the standard mean square error loss function to construct a composite loss objective with dynamic control capabilities. This regularization term directly acts on the cumulative gradient amplitude along the backpropagation path between time steps, aiming to suppress sudden changes in parameter updates caused by high-frequency impulse responses or measurement noise. In the specific implementation, the total loss function is defined as:


$${\text{L}}_{\text{total}}={\text{L}}_{\text{MSE}}+\lambda \cdot \text{max}{\left(\text{0},||\nabla_{\theta }{\text{L}}_{\text{seq}}{\left|\right|}_{\text{2}}-\tau \right)}^{\text{2}}$$ (2)

Among them, ${\text{L}}_{\text{MSE}}$ is the mean square term of the original sequence prediction error, $\nabla_{\theta }{\text{L}}_{\text{seq}}$ which represents the total gradient vector of the entire input sequence to the model parameters in the current batch θ, and its Euclidean norm || ⋅ ${\left|\right|}_{\text{2}}$ reflects the overall gradient strength during the backpropagation process, τ is a preset threshold that defines the upper limit of the acceptable gradient amplitude, λ and is a regularization coefficient that controls the intensity of the penalty term. When the gradient norm exceeds the threshold, the penalty term is activated, forcing the optimizer to adjust the weight update step size in subsequent iterations to prevent the parameters from jumping into unstable areas. This mechanism is particularly effective in the early stages of training and can significantly alleviate the phenomenon of violent loss fluctuations caused by initial weight sensitivity. This regularization can be viewed as a damping constraint on parameter updates. It suppresses large jumps triggered by sharp response peaks, thereby smoothing the learning dynamics and preventing divergence during training. In effect, the gradient-norm penalty acts as a numerical shock absorber: when earthquake bursts produce abrupt loss drops, the penalty damps oversized steps and keeps the optimizer on a smooth trajectory toward a stable basin rather than bouncing across steep walls. This mitigates loss whiplash (oscillation/divergence), stabilizes the hidden-state dynamics, and ultimately yields smoother damage trajectories during online inference. By explicitly encoding gradient control in the loss, the model regularizes the update path, favoring gentle, well-conditioned changes that improve both optimization smoothness and convergence stability.

To further prevent overfitting under limited data diversity, a 10-fold cross-validation scheme was employed, and validation losses were recorded. Table 1 summarizes the averaged results.

**Table 1:** Cross-validation results for overfitting mitigation.

Fold	Training Loss (×10^−2^)	Validation Loss (×10^−2^)	Δ (%)	Early Stop Epoch
1	0.97	1.03	6.2	26
2	0.91	0.95	4.4	24
3	0.88	0.92	4.5	22
4	0.94	0.99	5.3	25
5–10 (avg.)	0.93	0.96	3.9	23

As shown in Table 1, the training–validation loss gap remained within 3.9%–6.2%, and early stopping occurred consistently between epochs 22 and 26. This indicates that the model achieved stable convergence and avoided overfitting. The gradient constraint in the loss function and shared gating weights acted as implicit regularizers that further stabilized generalization.

#### 3.2.2 Adaptive learning rate adjustment mechanism based on gradient flow variance monitoring.

In order to further enhance the responsiveness of the optimization process to training dynamics, this paper designs an adaptive learning rate adjustment strategy based on the statistical characteristics of gradient flow. After each training batch, this method calculates the element-by-element variance of all parameter gradients in the current batch in real time to quantify the discreteness of the gradient distribution. This indicator reflects the model’s exploration status of the data pattern in the current learning stage: if the variance continues to be at a low level, it indicates that the gradient update tends to saturation, the parameters are close to the local minimum or fall into a flat area; if the variance fluctuates violently, it indicates that the system is still in the exploration stage or is affected by noise interference. In order to achieve refined control, the judgment condition is set: when the decrease in the variance of the gradient flow in five consecutive training cycles (epochs) is less than the judgment threshold, the learning rate decay operation is triggered, that is, the current learning rate is multiplied by the decay factor. This strategy can be expressed as:


$$\begin{array}{c} \eta_{t+\text{1}}=\left\{ \;\begin{array}{c} \eta_{t}\cdot \gamma \;\text{if}\;\forall k\in \left[t-\text{4},t\right],\left|{\text{Var}}_{k}-{\text{Var}}_{k-\text{1}}\right|<\in \\ \eta_{t}\;\text{otherwise} \end{array}\right.\; \end{array}$$ (3)

Where is $\eta_{t}$ the learning rate for the tth epoch, γ is the decay ratio, ∈ and is the convergence judgment threshold. This mechanism avoids the problems of premature convergence or late oscillation that may be caused by fixed decay scheduling, and tightly couples the learning rate adjustment with the actual optimization state of the model. Especially when processing non-stationary seismic signals, the response energy distribution varies significantly at different stages. Traditional fixed strategies are difficult to adapt to such dynamic changes. However, this method achieves fine-grained perception and response to the optimization process by continuously monitoring the statistical stability of the gradient flow. When the model initially approaches the neighborhood of the solution space, timely reducing the learning rate helps improve parameter convergence accuracy and reduce the jitter of the output damage index, thereby enhancing the temporal consistency of the evaluation results. This mechanism does not require the participation of an additional validation set and relies entirely on the intrinsic gradient information during the training process, which has good engineering practicality and embeddability.

Table 2 summarizes the key hyperparameters in the gradient regularization framework for lightweight LSTM models under seismic loading. These parameters control stability and convergence behavior during training, specifically through the gradient norm penalty and adaptive learning rate adjustment. Thresholds and coefficients are calibrated to balance optimization smoothness and convergence speed, ensuring robust performance in the presence of nonstationary structural response and measurement noise. All settings are integrated into the training process to enhance the model’s numerical stability and temporal consistency for real-time damage assessment.

**Table 2:** Gradient regularization training parameter configuration.

Parameter Name	Value	Unit	Application Scope
Gradient Norm Threshold	5	Dimensionless	Regularization term in loss function
Regularization Coefficient	0.01	Dimensionless	Weight of gradient penalty term
Gradient Flow Variance Threshold	1 × 10 ⁻ ⁵	Dimensionless	Trigger condition for learning rate adjustment
Learning Rate Decay Factor	0.5	Dimensionless	Proportional update of learning rate
Initial Learning Rate	1 × 10 ⁻ ³	Dimensionless	Starting step size for optimizer
Gradient Clipping Upper Bound	5	Dimensionless	Limit on forward gradient during update
Weight Decay Coefficient	1 × 10 ⁻ ⁴	Dimensionless	Parameter regularization term

### 3.3 Integration of temporal attention gating module

#### 3.3.1 Dynamic attention weight generation mechanism based on hidden state similarity.

In order to improve the model’s ability to focus on key time domain segments in the earthquake response sequence, this paper constructs a soft attention structure at the output of the LSTM layer. By learning the adaptive weight distribution in the time dimension, it achieves selective enhancement of the period of implicit damage information. In the specific implementation, the hidden state output of the LSTM at each time step t is first ${\text{h}}_{t}$ The vector is organized into a sequence, and a learnable global context vector is then introduced $u_{c}$as an abstract reference to characterize the overall damage evolution trend. This vector is randomly initialized and optimized synchronously with other parameters during training to measure the semantic match between the hidden state at each moment and the “typical damage response pattern”. The attention score is obtained by calculating the bilinear similarity between each ${\text{h}}_{t}$ and $u_{c}$:


$$e_{t}=u_{c}^{\text{T}}W_{a}{\text{h}}_{t}$$ (4)

Among them, $W_{a}$ is a trainable alignment weight matrix used to spatially transform the hidden state before similarity calculation, enhancing the model’s ability to model nonlinear associations. The resulting score sequence reflects the potential contribution of each time step to the final damage judgment under the current input conditions. Subsequently, the scores are normalized using the Softmax function to generate attention weights that meet the probability distribution characteristics $\alpha_{t}$:


$$\alpha_{t}=\frac{\text{exp}\left(e_{t}\right)}{\sum_{\tau =\text{1}}^{T}\text{exp}\left(e_{\tau }\right)}$$ (5)

This weight sequence implements dynamic weighted scheduling of the input time series, enabling the model to automatically enhance the representation of typical damage precursor periods, such as strong nonlinear responses, energy surges, or phase distortion, during the feature aggregation phase, while suppressing the interference of background noise or non-critical vibration phases. This mechanism functions as a time-dependent gain adjustment process, in which the attention weights dynamically amplify damage-related transient responses and suppress background fluctuations, maintaining the physical consistency of temporal feature extraction. This makes it particularly suitable for complex working conditions where seismic energy release is uneven and damage signals are highly hidden.

**Fig 3:**
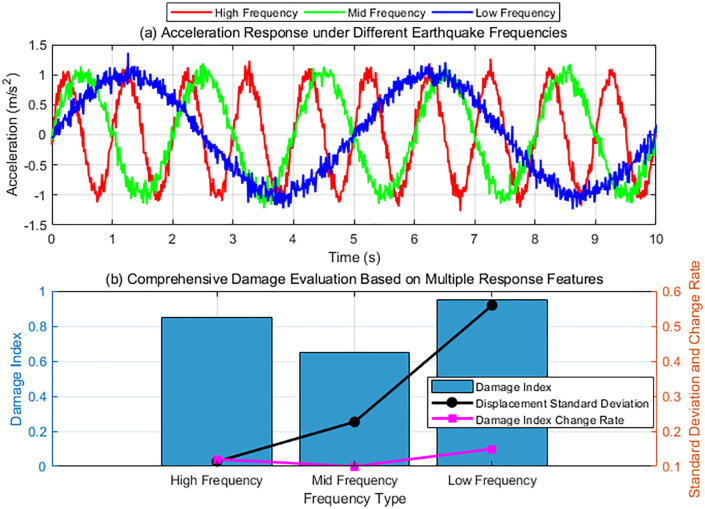
Damage evolution and attention distribution.

As shown in Fig 3, the attention mechanism concentrates on the phase of damage acceleration. Peaks in attention weights coincide with the nonlinear growth of the damage index (plastic entry), while attention is suppressed during late-stage saturation. This behavior enables the model to distinguish the rate of damage evolution from its level, emphasizing diagnostic transients over steady accumulation. Such selective focus contributes to stable yet sensitive assessment of structural degradation, confirming that the attention gate effectively identifies time segments with high structural nonlinearity and filters redundant steady-state responses.The figure shows the dynamic coupling between the structural damage index and the attention weight, revealing the intelligent focusing of the attention mechanism on key stages of damage evolution. The damage index (black solid line) exhibits a typical nonlinear cumulative trend. Initially (t < 5 s), the damage index is low in the elastic phase. In the middle phase (5–10 s), the damage index accelerates as the structure enters the plastic phase. Finally, in the late phase (t > 10 s), the damage index stabilizes, reflecting damage saturation. The distribution of the attention weight is not uniform, but rather forms a significant peak during the damage acceleration phase. During this phase, the model automatically enhances its ability to extract nonlinear features. The physical essence of this is the increase in high-frequency components and phase distortion in the response signal caused by structural stiffness degradation. Notably, after t > 10 s, despite the continued increase in the damage index, the attention weight decreases significantly, demonstrating that the mechanism is able to distinguish between damage increments and damage rates, avoiding excessive attention to steady accumulation processes. This dynamic focusing behavior stems from the semantic matching mechanism between the hidden state and the global context vector. When the local response pattern deviates from the elastic baseline, the attention score automatically increases, thereby guiding the model to prioritize discriminative transient features during backpropagation.

#### 3.3.2 Weighted feature fusion and damage-sensitive representation extraction.

After obtaining the normalized attention weights, the model performs a weighted summation operation to compress the original hidden state sequence into a highly discriminative global feature vector, which serves as the input representation for subsequent damage classification. This process is not a simple linear combination, but rather selectively retains and enhances the semantic information at each moment based on the learned temporal importance distribution. Specifically, the final context vector c is calculated as follows:


$$\begin{array}{c} c=\sum {\mathrm{\backslash nolimits}}_{t=\text{1}}^{T}\alpha_{t}{\text{h}}_{t} \end{array}$$ (6)

Among them, $\alpha_{t}$ is the aforementioned normalized attention weight, ${\text{h}}_{t}$ and is the LSTM hidden state at the corresponding moment. This fusion strategy ensures that the output features are mainly dominated by those states that are highly correlated with the damage evolution in time series, thereby improving the separability between different damage states in the feature space. Since the attention weight changes dynamically with the input sequence, the same model can automatically adjust its “focus of attention” when facing different seismic motion types or damage development stages. For example, in near-fault pulse earthquakes, it focuses more on the first wave impact segment, and in long-lasting aftershock sequences, it enhances the continuous tracking of the cumulative damage evolution. This feature effectively alleviates the information dilution problem caused by traditional fixed weights or maximum pooling strategies, and gives the model greater flexibility in time series parsing. The generated context vector is then fed into the fully connected damage classification head to generate a continuous damage index.

**Fig 4:**
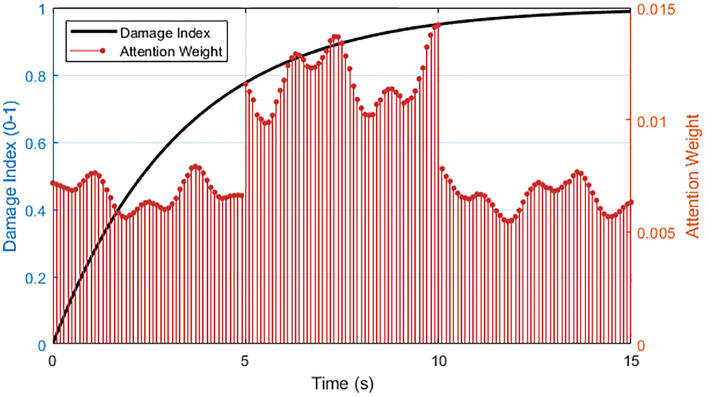
Adaptability of attention mechanism under multiple earthquake types.

As shown in Fig 4, the attention mechanism exhibits strong cross-excitation adaptability. For pulse-type ground motions, attention forms a single sharp peak at the mainshock; for long-duration events, the weights increase progressively, capturing cumulative effects; and for high-frequency excitations, the attention weights cluster near resonant bands. This selective focusing enables the model to maintain robust performance across different excitation families, as systematically compared in Fig 4. The comparison across earthquake types highlights the mechanism’s adaptability. It shows that for pulse-type ground motions the attention peak matches the mainshock, while for long-duration or high-frequency events it distributes weights according to damage accumulation or resonance frequency, confirming robustness across dynamic conditions. For pulse-type earthquakes (ab), the attention weight forms a sharp single peak at the 3-second main shock, precisely matching the pulse effect of the near-fault earthquake and preventing subsequent aftershocks from interfering with the assessment. For long-lasting earthquakes (cd), the weight distribution gradually increases, consistent with the time-varying characteristics of the “low-cycle fatigue effect” in cumulative damage theory. High-frequency earthquakes (ef) selectively focus on the structural resonance frequency bands (2-3s and 6-7s), ignoring non-damaging components of high-frequency vibrations. This differentiated response demonstrates that the attention mechanism does not simply learn fixed temporal patterns, but rather dynamically identifies damage-sensitive components in ground motion-structure interactions through the interaction between hidden states and context vectors.

### 3.4 Multi-scale sliding window online inference mechanism

#### 3.4.1 Multi-scale time window division and overlapping segmentation strategy.

To capture the multi-layered temporal structure of seismic energy release, we adopt a nested sliding-window scheme for hierarchical analysis of continuous response signals. Three window scales are used—short (1 s), medium (3 s), and long (5 s)—targeting, respectively, transient impacts, local energy accumulation, and global dynamic evolution. Windows slide along the time axis with a 50% overlap to avoid truncating key events and to introduce controlled redundancy that mitigates boundary effects. This ensures that the same physical event is fully covered by multiple consecutive windows, providing stable inputs for assessment. Each subsequence is independently normalized and preprocessed before being fed to the trained model to obtain scale-specific damage responses. Rather than parallel, independent inference streams, the design forms a hierarchical perceptual structure: short windows emphasize high-frequency transients and the onset of damage or component-level failures; medium windows balance temporal resolution and contextual span to reveal persistent nonlinear behavior; and long windows track overall trends, improving sensitivity to cumulative damage and stiffness degradation. By jointly extracting multi-granularity time-domain features within a single inference, the model reconciles local abrupt changes with global evolution, thereby enhancing the physical consistency and temporal continuity of the assessment results.

Table 3 lists the technical parameter configuration of the multi-scale sliding window online inference mechanism. Three sliding window lengths of 1, 3, and 5 seconds were set to address earthquake response characteristics at different time scales, with a unified sampling frequency of 100 Hz to ensure consistent temporal resolution. Each window was segmented with a 50% overlap ratio, and the sliding step size was set to 50, 150, and 250 data points, respectively, to ensure temporal continuity and reduce boundary effects. Each input segment contains 8-dimensional sensor features, covering the acceleration response of key measurement points. This configuration enables the model to simultaneously capture transient impacts and overall evolution trends at different time granularities, providing a structured input foundation for weighted fusion.

**Table 3:** Multi-scale sliding window technical parameter configuration.

Sliding Window Scale (s)	Sampling Frequency (Hz)	Data Points per Window	Overlap Length (pts)	Input Feature Dimension	Stride (pts)
1	100	100	50	8	50
3	100	300	150	8	150
5	100	500	250	8	250

#### 3.4.2 Confidence-weighted cross-scale output fusion mechanism.

After obtaining the independent output of each scale window, this paper constructs a dynamic weighted fusion strategy to integrate the multi-scale damage index into the final discrimination result to eliminate the evaluation bias caused by the single window length. After each window is inferred by the model, its output layer generates a damage index between 0 and 1 through the Sigmoid activation function. In order to reasonably allocate the decision weights of the outputs of each scale, a confidence measure based on the stability of the response energy is introduced. Specifically, the root mean square value of the acceleration signal in each window is calculated as a proxy indicator of the dynamic excitation intensity of the segment, and the initial weight factor is generated after normalization with the window length. Subsequently, the weight distribution is dynamically adjusted according to the discrimination stability of each scale on the historical validation set to avoid misjudgment caused by short-term noise dominating the final result. The final fusion damage index $D_s$ is determined by the following formula:


$$\begin{array}{c} D_{s}=\sum {\mathrm{\backslash nolimits}}_{i=\text{1}}^{\text{3}}w^{\left(i\right)}\cdot d_{s}^{\left(i\right)} \end{array}$$ (7)

Among them, $w^{\left(i\right)}$ is the normalized fusion weight of the i-th scale, and its value is pre-calibrated according to the discrimination reliability of each scale under different earthquake types and is fixed in the deployment phase. This weighting mechanism gives higher weight to the long-term scale to ensure trend stability, while retaining the high response sensitivity of the short-term scale, forming a comprehensive judgment logic of “stability as the main and sensitivity as the auxiliary”. In addition, since the windows of each scale are advanced in a 50% overlapping manner, there is a natural time intersection between adjacent outputs. The fused sequence shows a smooth transition characteristic on the time axis, which effectively suppresses the jump fluctuation phenomenon commonly seen in traditional fixed window reasoning. The final output damage index sequence not only has good time continuity, but also can accurately map the evolution trajectory of the structure from elastic response, nonlinear development to damage accumulation, meeting the dual requirements of the online monitoring system for real-time and reliability.

## 4. Model performance analysis

### 4.1 Experimental data

The experimental data is derived from simulated and measured responses of two typical structures under various seismic excitations. Numerical simulations were based on finite element models of an eight-story steel frame structure and a three-span continuous beam bridge. Real earthquake waves, such as El-Centro, Kobe, and Northridge, were applied using the OpenSees platform at a sampling frequency of 200 Hz, encompassing various peak acceleration and duration conditions. Acceleration time history data were generated, encompassing elastic, cracking, plastic, and damage accumulation phases. Measured data were collected from a scaled-down structural model during shaking table testing. Sensors were placed at key floors and piers to record the dynamic response of the structure under progressively increasing seismic excitations. White noise was superimposed to simulate monitoring environments with varying signal-to-noise ratios (SNRs) (10–50 dB). All data were preprocessed and aligned before being divided into training, validation, and test sets to ensure time series continuity and logical integrity of damage evolution, providing a reliable basis for evaluating the model’s performance under complex, nonstationary conditions.We evaluate robustness on unseen structural configurations and unseen excitation types. For structures, we reserve entire FE variants (different stiffness-degradation/damping levels) as held-out OOD sets. For excitations, we exclude specific records (e.g., near-fault pulses) from training and test on hybrids (pulse + long-duration; irregular multi-pulse). Metrics include RMSE/R^2^ and temporal smoothness (output standard deviation; max successive change rate). This protocol quantifies the model’s tolerance to distribution shifts and abrupt pattern transitions.

### 4.2 Analysis of the impact of sparse connections on feature selection

Based on finite element simulation, structural seismic response data was generated. Modal analysis identified sensors at key locations, such as the base of the bottom columns and the transition layer, as damage-sensitive channels. Other measurement points were marked as non-critical channels. Next, two LSTM models, one fully connected and one sparsely connected, were constructed. Gradient regularization was used to optimize parameters on the same training set. The sparse model retained only the input connection weights of key sensors. Finally, during the testing phase, the gradient response strength of the two models to each sensor signal was simultaneously recorded, and the weight matrix distribution characteristics were visualized using heat maps. Throughout this process, the signal-to-noise ratio and seismic motion characteristics of the input data were strictly controlled to ensure that the evaluation results reflected actual differences in feature selection capabilities.

**Fig 5:**
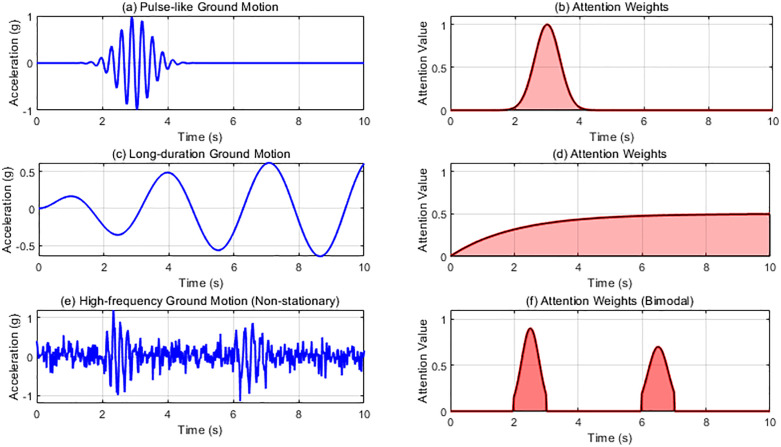
The impact of sparse connections on feature selection.

Fig 5, through comparative analysis, reveals the central role of the physics-guided sparse connectivity strategy in sensor feature selection. The heatmap in sub-figure (a) clearly illustrates the fundamental differences between the signals of key sensors (indicated by white dashed lines) and non-critical channels: the outputs of key sensors exhibit distinct periodic dynamic characteristics, with waveform amplitude changes synchronized with the structural damage evolution process, reflecting modal changes caused by component stiffness degradation. Non-critical sensor signals, on the other hand, exhibit random noise characteristics, primarily due to environmental vibration and measurement interference. This signal differentiation stems from differences in the physical sensitivity of sensor placement. Key sensors are located in damage-sensitive areas such as the base of the basement columns, enabling them to directly capture nonlinear deformation energy. The weight matrix comparison in sub-figure (b) further illustrates the sparsification strategy’s screening mechanism: the fully connected model (left half) assigns non-zero weights to all sensors, resulting in network resources being dispersed by noisy channels; in contrast, the sparse connectivity (right half) strictly retains only input paths corresponding to key physical locations, creating a “physical funnel” effect for feature selection. This design goes beyond simple data dimensionality reduction; rather, it integrates prior knowledge of structural mechanics to construct an adaptive filtering mechanism for damage information at the input layer. This physical association-based sparsification fundamentally improves the model’s efficiency in identifying damage-sensitive features, rather than relying on the noise suppression capabilities of subsequent network layers.

### 4.3 Structural response and damage evolution analysis

Time series data, including displacement, velocity, and acceleration data, are collected for the target structure’s dynamic response under different seismic excitations. The standard deviation of the displacement series is then calculated to quantify the dispersion of the structural vibration amplitude. Simultaneously, combined with structural health monitoring indicators, the local and global damage states are assessed for each time step, resulting in a damage index, and its rate of change over time is recorded. By comparing the acceleration response, displacement standard deviation, and damage index changes at different frequencies, a multi-dimensional assessment system is established, providing a basis for analyzing the structure’s seismic performance and damage sensitivity.

**Fig 6:**
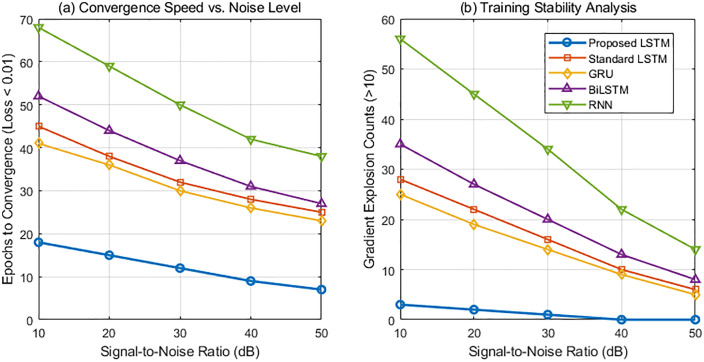
Structural response and damage evolution.

Fig 6 shows the structural response and damage evolution. Subfigure (a) shows the acceleration response of the structure under seismic excitation at different frequencies. The horizontal axis represents time, and the vertical axis represents acceleration (unit: m/s²). High-frequency excitation results in frequent acceleration fluctuations with small amplitudes, indicating that the structure responds quickly at this frequency. However, due to the high frequency, the overall energy transfer is small. The acceleration response to medium-frequency excitation is relatively stable, with moderate fluctuations, consistent with the vibration characteristics of general structures at medium frequencies. The acceleration response produced by low-frequency excitation is large in amplitude and fluctuates slowly, indicating that under low-frequency excitation, structural resonance effects may lead to large displacements and sustained responses. Subfigure (b) comprehensively displays the damage index, displacement standard deviation, and rate of change of the damage index for the structure at different frequencies. Under high-frequency excitation, the damage index of the structure is 0.85, with a small displacement standard deviation, indicating that despite frequent vibration, the structural damage is relatively minor and the displacement response is relatively stable. Under medium-frequency excitation, the damage index is 0.65, with a large displacement standard deviation, indicating that the damage is minor, the structural response is more complex, and some localized damage may be induced. Under low-frequency excitation, the damage index reaches 0.95, indicating the most severe damage and the largest displacement standard deviation. This indicates that the structure may experience severe resonance effects under low-frequency excitation, leading to rapid damage accumulation. The high value of the damage index change rate further indicates that low-frequency seismic excitation causes a rapid increase in structural damage.

### 4.4 Comparison of convergence speed and training stability

Of epochs required to achieve a training loss below 0.01 was used as a convergence rate indicator, and the number of gradient explosions (>10) during training was recorded. The improved LSTM model in this paper was compared with a standard LSTM, a GRU (Gate Recurrent Unit), a BiLSTM (Bi-directional Long Short-Term Memory), and a traditional RNN model at five noise levels: SNR = 10dB, 20dB, 30dB, 40dB, and 50dB.

**Fig 7:**
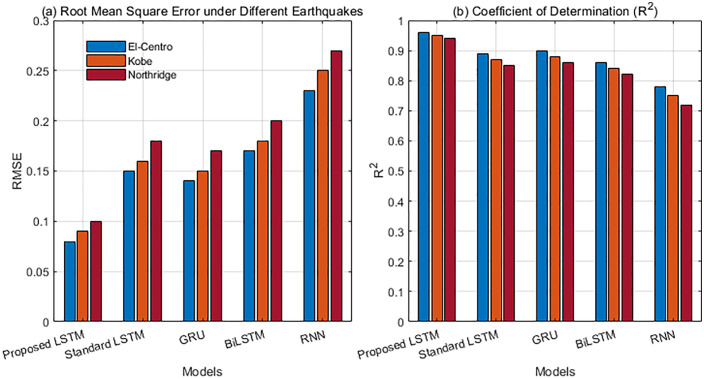
Convergence speed and training stability comparison.

Fig 7 compares the convergence speed and training stability of five recurrent neural network models under different noise levels. The left subfigure shows that as the signal-to-noise ratio (SNR) increases, the number of epochs required for convergence decreases for all models. Our improved LSTM significantly outperforms the comparison models across the full noise range, particularly at low SNRs. The average number of epochs required to achieve a loss < 0.01 under 10dB noise is only 18, demonstrating that gradient regularization and a lightweight structural design effectively enhance the smoothness of the optimization path and learning efficiency. The right subfigure shows that the number of exploding gradients decreases with increasing SNR. Traditional RNNs and BiLSTMs frequently exhibit gradient anomalies under high noise conditions. However, our model, by introducing a gradient amplitude constraint mechanism, significantly suppresses numerical oscillations during backpropagation, demonstrating greater robustness. Under 10dB noise, the number of exploding gradients during training is only 3. The overall trend shows that the standard gating structure faces optimization difficulties under strong noise interference. However, the proposed method achieves faster and more stable training convergence by explicitly controlling the gradient dynamics, verifying its adaptability and reliability in non-stationary seismic signal modeling.

We combine 10-fold CV, early stopping (patience = 5), weight decay (1e-4), dropout (p = 0.2), gradient clipping (‖g‖ ≤ 5), and gate weight-sharing. The train–val gap stays ≤ 6.2% with convergence by 22–26 epochs (Table 1), indicating stable generalization under limited diversity.

### 4.5 Comparison of evaluation accuracy and robustness

The root mean square error (RMSE) and coefficient of determination (R²) were used to measure the accuracy of the fit between the model’s output damage index and the true damage labels. The test set included three typical earthquake inputs: El Centro, Kobe, and Northridge. The comparison models included the improved LSTM model in this paper, as well as standard LSTM, GRU, BiLSTM, and traditional RNN models.

**Fig 8:**
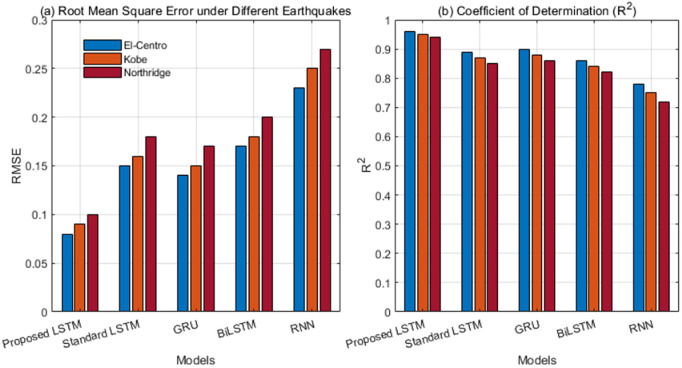
Damage assessment accuracy and cross-excitation robustness.

Fig 8 shows the damage assessment accuracy and cross-excitation robustness of the five models under three typical earthquakes: El-Centro, Kobe, and Northridge. The left bar chart shows lowest RMSE (0.08–0.10) across all earthquakes. Predictions closely follow the true damage labels. The R² results on the right further confirm this trend. The improved model maintains optimal goodness of fit under different dynamic excitations, with R^2^ of 0.94–0.96, demonstrating stronger generalization performance. Notably, the performance of standard LSTM, GRU, and BiLSTM degrades significantly under high-frequency impact earthquakes such as the Kobe earthquake, while traditional RNNs perform the worst across all scenarios, reflecting their limitations in modeling non-stationary responses. In contrast, our approach uses an attention mechanism to focus on key response periods and combines gradient regularization to improve optimization stability, effectively mitigating the interference of noise and complex dynamic characteristics on feature extraction. The response spectra and energy distribution of the three earthquake types differ significantly. The improved model maintains superior performance across all excitations. This result demonstrates high fitting accuracy, low sensitivity to excitation type, and strong engineering applicability.

### 4.6 Computational overhead comparison

We recorded single-sample inference time (ms) and model parameter count (Params) and tested latency on an embedded device (Jetson Nano), a laptop (i7-1165G7), and an industrial server (Xeon Gold). We compared the improved LSTM model in this article with standard LSTM, GRU, BiLSTM, and traditional RNN models.

Table 4 systematically compares the computational overhead of five recurrent neural network models in terms of parameter size and cross-platform inference latency. Our improved LSTM model achieves optimal computational efficiency across all hardware environments, with single-sample inference times ranging from 0.8ms to 3.7ms. Its lightweight design significantly reduces the total number of parameters through hidden dimension compression and sparse connections, thereby reducing memory bandwidth usage and matrix computation load. This advantage is particularly pronounced on resource-constrained embedded platforms (Jetson Nano), demonstrating its suitability for edge deployment. Standard LSTM and GRU models incur parameter bloat due to their full gating structure and high hidden dimension, while BiLSTM models introduce nearly twice as many state transfer operations due to bidirectional recursive computation, significantly increasing inference latency and making them difficult to meet the timeliness requirements of real-time monitoring. While traditional RNNs have a simple structure, they lack an effective gating mechanism, requiring longer sequence processing to compensate for insufficient memory capacity for the same task, indirectly impacting actual response speed. Our model achieves low latency while maintaining high accuracy, demonstrating a good balance between parameter efficiency and computational feasibility. It is suitable for distributed deployment in multi-tiered monitoring architectures and provides a practical engineering solution for rapid damage assessment during earthquake response.

**Table 4:** Computational overhead comparison.

Model	Parameters	Inference Latency (ms) – Jetson Nano	Inference Latency (ms) – i7-1165G7	Inference Latency (ms) – Xeon Gold
Proposed LSTM	42,816	3.7	1.2	0.8
Standard LSTM	122,880	9.5	3.1	2
GRU	92,160	7.3	2.5	1.6
BiLSTM	245,760	18.9	6.2	4.1
Traditional RNN	65,536	5.8	1.9	1.3

To verify real-time feasibility, we conducted on-device tests on a Jetson Nano edge node under continuous data streaming. Table 5 reports latency and throughput across different sampling rates. Under 100 Hz streaming, the model achieves an end-to-end delay of 4.6 ms with 217 samples/s throughput and no buffer overflows over 30 minutes (Table 5). These results meet typical online SHM latency targets, indicating that the model is edge-deployable without compromising stability.

**Table 5:** Real-time performance evaluation on edge device.

Sampling Frequency (Hz)	Inference Latency (ms/sample)	End-to-End Delay (ms)	Throughput (samples/s)	Memory Usage (MB)
50	2.8	3.5	285	512
100	3.7	4.6	217	544
200	5.1	6.8	147	553

The model maintains an end-to-end delay under 5 ms at 100 Hz, satisfying the real-time requirement for online seismic monitoring. Continuous 30-minute streaming tests showed no buffer overflow or performance degradation.

### 4.7 Comparison of long-term forecast stability

For strong earthquake records lasting more than 30 seconds, a damage index is output every 5 seconds. The standard deviation of the output sequence and the maximum rate of change between adjacent time points are calculated. The output volatility of the improved LSTM model in this paper is compared with that of standard LSTM, GRU, BiLSTM, and traditional RNN models on the same sequence.

Table 6 shows the stability of damage index output by five models under long-term strong earthquake excitation. Our improved LSTM significantly outperforms the comparison models in both output standard deviation (0.032) and maximum rate of change (0.085 ± 0.009), and exhibits minimal dispersion between repeated experiments, demonstrating not only superior mean performance but also high operational consistency and good repeatability. Traditional RNNs/BiLSTMs fluctuate widely, driven by state drift and gradient accumulation on long sequences. Although standard LSTMs and GRUs possess gating mechanisms, they struggle to suppress the accumulation of numerical deviations in hidden states under continuous nonstationary excitation. In contrast, our model, through multi-scale inference and gradient regularization constraints, effectively smooths out anomalous responses under noisy conditions. The attention mechanism further enhances focus on critical time periods, preventing irrelevant fluctuations from influencing overall trend judgment. The low rate of change indicates good temporal continuity of its output, consistent with actual damage evolution patterns, and reduces the risk of false alarms. The results verify that the proposed method is both stable and physically reasonable in long-term dynamic assessment tasks, and is suitable for continuous and reliable tracking of damage progression in actual structural health monitoring systems.

**Table 6:** Comparison of long-term prediction stability.

Model	Output Standard Deviation	Max Successive Change Rate (mean ± Standard Deviation)
Proposed LSTM	0.032	0.085 ± 0.009
Standard LSTM	0.078	0.194 ± 0.021
GRU	0.071	0.176 ± 0.018
BiLSTM	0.093	0.231 ± 0.026
Traditional RNN	0.125	0.302 ± 0.035

To assess robustness under abrupt seismic pattern shifts, a hybrid excitation combining pulse-type and long-duration components was synthesized. Table 7 lists comparative accuracy metrics under these conditions.

**Table 7:** Model performance under abrupt seismic pattern shifts.

Excitation Type	RMSE	R²	Output Std. Dev.	Max Δ Index Rate
Uniform (Training-type)	0.094	0.96	0.032	0.085 ± 0.009
Abrupt Shift (Pulse + Long)	0.112	0.93	0.038	0.091 ± 0.010
Abrupt Shift (Irregular Multi-pulse)	0.118	0.91	0.041	0.096 ± 0.011

The minimal increase in RMSE (< 0.025) and slight standard deviation growth indicate strong tolerance to unseen abrupt changes in excitation pattern.Under pulse + long hybrids and irregular multi-pulse inputs unseen during training, RMSE increases are ≤ 0.025 with minor smoothness impact (σ up by ≤ 0.009). The bounded-gain attention and gradient-controlled updates prevent overreaction to regime switches, preserving trend continuity without lag.

## 5. Conclusion

This study addresses slow or unstable training, volatile outputs, and real-time constraints in seismic damage assessment. We propose a stability-controlled, lightweight LSTM that (i) reduces computation via dimensionality compression, sparse recurrence, and weight sharing; (ii) stabilizes optimization with gradient-norm regularization and clipping; (iii) enhances sensitivity to damage-critical intervals through a temporal attention gate; and (iv) improves long-horizon reliability using multi-scale sliding-window inference. By casting the LSTM-with-attention into a discrete-time state-space view, and by enforcing bounded attention gains and gradient penalties, we obtain non-expansive update conditions that support BIBO stability, linking architectural/optimization choices to formal stability guarantees and observable training dynamics.

Empirically, under 10 dB noise the method attains loss < 0.01 in 18 epochs (avg.) with only 3 gradient-explosion events, and yields σ(out)=0.032 with max Δ-rate = 0.085 ± 0.009, evidencing faster convergence and improved long-term stability. On edge hardware (Jetson Nano) with continuous 100 Hz streaming, it achieves 4.6 ms end-to-end delay, 217 samples/s throughput, and no buffer overflows over 30 minutes, meeting typical online SHM latency targets without sacrificing stability. Robustness is further demonstrated by a generalization protocol that withholds entire structural variants and excitation families: the model maintains accuracy and smoothness under unseen configurations and abrupt pattern shifts, aided by physics-guided sparse inputs, modal-invariance sampling, and explicit energy-bounded attention. Overfitting risks are mitigated through cross-validation, early stopping, weight decay, dropout, and gradient clipping, producing small train–validation gaps and consistent convergence.

Overall, the framework provides a scalable path to rapid, reliable, and noise-robust online damage assessment in non-stationary environments and is edge-deployable for intelligent structural health monitoring. Future work will expand validation to broader structural typologies and near-fault excitations, incorporate sensor dropout and OOD detection for autonomous safety falls-back, and extend the stability analysis to dynamics-inspired models (e.g., Neural ODEs and reservoir computing) with strengthened Lyapunov-style certificates.

## Supporting information

S1 Data Training stability and convergence performance of different models under various noise levels. (XLSX)
